# *In vivo* coherent Raman imaging of the melanomagenesis-associated pigment pheomelanin

**DOI:** 10.1038/srep37986

**Published:** 2016-11-28

**Authors:** Hequn Wang, Sam Osseiran, Vivien Igras, Alexander J. Nichols, Elisabeth M. Roider, Joachim Pruessner, Hensin Tsao, David E. Fisher, Conor L. Evans

**Affiliations:** 1 Wellman Center for Photomedicine, Harvard Medical School, Massachusetts General Hospital, 149 13^th^ Street, Charlestown, Massachusetts 02129, USA; 2 Harvard-MIT Division of Health Sciences and Technology, 77 Massachusetts Avenue E25-519, Cambridge, Massachusetts 02139, USA; 3 Cutaneous Biology Research Center, Harvard Medical School, Massachusetts General Hospital, 149 13^th^ Street, Charlestown, Massachusetts 02129, USA; 4 Harvard University Program in Biophysics, Building C2 Room 112, 240 Longwood Avenue, Boston, Massachusetts 02115, USA; 5 Ludwig Center at Harvard, Harvard Medical School, 200 Longwood Avenue, Boston, Massachusetts 02115, USA

## Abstract

Melanoma is the most deadly form of skin cancer with a yearly global incidence over 232,000 patients. Individuals with fair skin and red hair exhibit the highest risk for developing melanoma, with evidence suggesting the red/blond pigment known as pheomelanin may elevate melanoma risk through both UV radiation-dependent and -independent mechanisms. Although the ability to identify, characterize, and monitor pheomelanin within skin is vital for improving our understanding of the underlying biology of these lesions, no tools exist for real-time, *in vivo* detection of the pigment. Here we show that the distribution of pheomelanin in cells and tissues can be visually characterized non-destructively and noninvasively *in vivo* with coherent anti-Stokes Raman scattering (CARS) microscopy, a label-free vibrational imaging technique. We validated our CARS imaging strategy *in vitro* to *in vivo* with synthetic pheomelanin, isolated melanocytes, and the *Mc1r*^*e/e*^, red-haired mouse model. Nests of pheomelanotic melanocytes were observed in the red-haired animals, but not in the genetically matched *Mc1r*^*e/e*^*; Tyr*^*c/c*^ (“albino-red-haired”) mice. Importantly, samples from human amelanotic melanomas subjected to CARS imaging exhibited strong pheomelanotic signals. This is the first time, to our knowledge, that pheomelanin has been visualized and spatially localized in melanocytes, skin, and human amelanotic melanomas.

The yearly global incidence of melanoma is over 232,000 individuals, with more than 55,000 of those diagnosed succumbing to the disease^1^. Individuals with fair skin and red hair exhibit the highest risk for developing melanoma^2^, with evidence suggesting the red/blond pigment known as pheomelanin may elevate melanoma risk through both UV radiation-dependent and -independent mechanisms. Moreover, red-haired melanoma patients often develop amelanotic lesions, so-called due to the absence of macroscopically detectable dark eumelanin pigments within the visible tumour margin. Because they are harder to recognize upon examination, these tumours are frequently diagnosed at more advanced stages and are associated with higher mortality^3^. Similar amelanotic melanomas arise in mice bred on the *Mc1r*^*e/e*^ genetic background, which recapitulates the red-haired, fair-skinned phenotype. The non-visible pheomelanin in these lesions was found to functionally contribute to melanoma formation, as the introduction of an albino allele onto the same genetic background abrogated melanoma risk^4^. Although the ability to identify and monitor pheomelanin within skin is vital to improve our understanding of the underlying biology of these lesions, no tools exist for real-time, *in vivo* characterization of the pigment.

Studies using Raman spectroscopy to explore the pigment of red bird feathers previously suggested that a unique vibrational band centred between 2000–2100 cm^−1^ may be a marker for pheomelanin^5^,^6^. However, it was not clear if this weak vibrational resonance could be used for *in situ* identification of the red pigment. Coherent anti-Stokes Raman scattering (CARS) microscopy, a label-free vibrational imaging technique based on Raman scattering, offers a significantly enhanced signal level compared to spontaneous Raman scattering, and may be suitable for non-invasively identifying pheomelanin inside the skin in real time. Here we present the distribution of pheomelanin in cells and tissues can be visually characterized non-destructively and noninvasively *in vivo* with CARS microscopy. We validated our CARS imaging strategy *in vitro* to *in vivo* using synthetic pheomelanin, isolated melanocytes, and the *Mc1r*^*e/e*^, red-haired mice model. Nests of pheomelanotic melanocytes were observed in the red-haired animals, but not in the genetically matched *Mc1r*^*e/e*^*; Tyr*^*c/c*^ (“albino-red-haired”) mice. Importantly, samples from human amelanotic melanomas imaged with CARS microscopy exhibited strong pheomelanotic signals. This is the first time, to our knowledge, that pheomelanin has been visualized and spatially localized in melanocytes, skin, or human amelanotic melanomas.

## Results and Discussion

### Synthetic pheomelanin

To demonstrate that CARS microscopy can selectively and sensitively visualize pheomelanin, the pigment was synthesized following an established procedure that makes use of the mushroom tyrosinase enzyme^7^. When distributed as small particles in a water/hexane emulsion, synthetic pheomelanin was found to yield a very strong CARS signal whose vibrational spectrum corresponds to that observed with Raman spectroscopy (Supplementary Fig. S1)^5^,^6^.

### Pheomelanin in FACS-sorted mouse melanocytes

With the ability to visualize and spectroscopically confirm the presence of pheomelanin using CARS microscopy, the next step was the detection of naturally-occurring pheomelanin. Red-haired mice (*Mc1r*^*e/e*^) were bred to incorporate B6.Cg-*Gt(ROSA)26Sor*^*tm6(CAG-ZsGreen1)Hze*^ (Jackson Laboratory catalogue #007906) or B6.Cg-*Gt(ROSA)26Sor*^*tm9(CAG-tdTomato)Hze*^ (Jackson Laboratory catalogue #007909) and melanocyte targeted CRE (Tyr-CRE)^8^, thereby enabling FACS-based isolation of neonatal ZsGreen or tdTomato fluorescent tagged melanocytes. A detailed protocol regarding melanocyte extraction can be found in the Methods section. The tdTomato mice were sacrificed, their skin harvested, and dermal cells sorted based on fluorescence of both tdTomato and FITC-labelled antibodies tagging c-Kit, a surface marker^9^. ZsGreen-labelled mice underwent a similar procedure, with melanocytes selected via their ZsGreen and c-Kit signals. The sorted melanocytes were fixed, and imaged with both CARS and confocal fluorescence microscopy.

**Figure 1:**
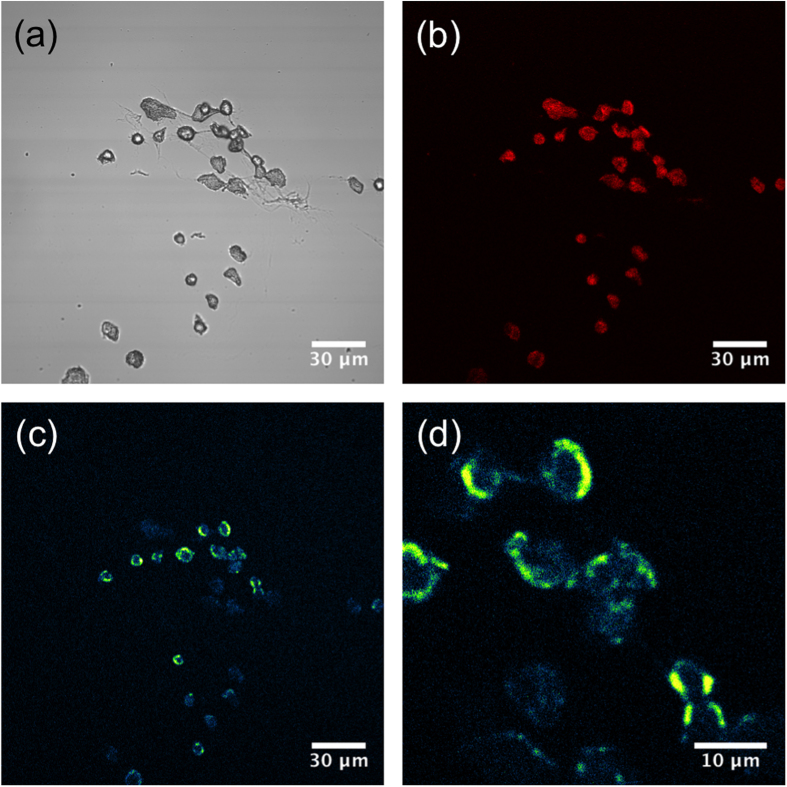
Melanocytes isolated from red-haired C57BL/6 (*Mc1r*^*e/e*^, *Tyr*^+/+^) mice exhibit strong CARS signal at ω_P_ − ω_S_ = 2000 cm^−1^. (**a**) Trans-illumination image acquired with the pump beam, where the overall shape of the cells can be well visualized. (**b**) Confocal fluorescence image of tdTomato. (**c**) False colour (“Green fire blue” colour in ImageJ) CARS image mapping intracellular pheomelanin distribution. (**d**) 4X-zoomed view of (**c**) showing a perinuclear distribution of signal intensity, consistent with the known biology of protective melanin caps.

As shown in Fig. 1, pheomelanin signals exhibited the predicted perinuclear localization of melanosomes. As a control, genetically matched tdTomato-tagged, tyrosinase-deficient (*Mc1r*^*e/e*^, *Tyr*^*c/c*^) “albino-red-haired” mouse-derived melanocytes were subjected to the same analysis and no visible pheomelanin signals were observed (Supplementary Fig. S2). Importantly, CARS spectra acquired from the red-haired mice, but not the albino red-haired mice, matched CARS spectra collected from synthetic pheomelanin, confirming the ability of coherent Raman imaging to selectively visualize the red pigment within cells.

### *Ex vivo*, *in vivo*, and histological visualization of pheomelanin in mouse skin

**Figure 2:**
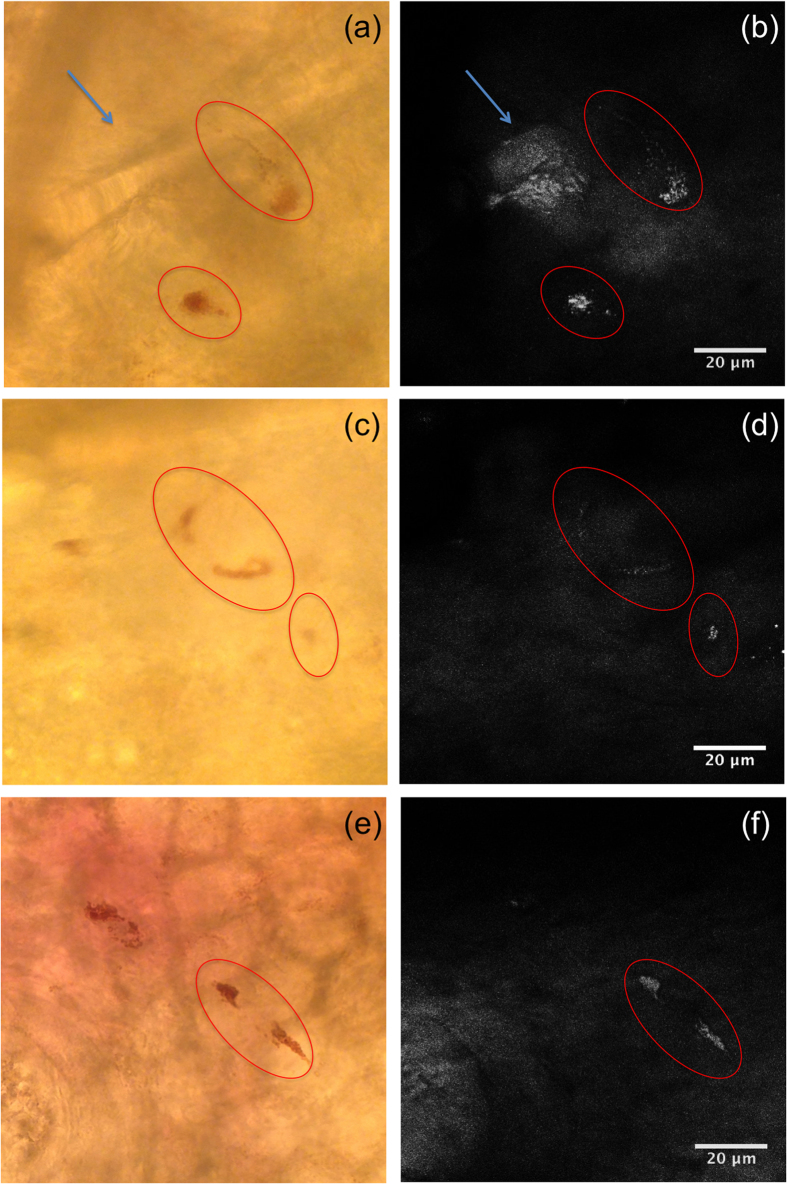
Imaging of pheomelanin stores in red-haired mouse ear skin. (**a**,**c**,**e**) Brightfield trans-illumination image acquired from the microscope eyepiece. (**b**,**d**,**f**) Maximal projection view of CARS image stack of the mouse ear, showing bright granules from the pheomelanin stores (red circles) within melanocytes and at the base of the hair follicle. A CARS image stack acquired with the pump beam set to 871 nm (ω_P_ − ω_S_ = 1866 cm^−1^) was subtracted from the image stack acquired with the pump beam set to 861 nm (ω_P_ − ω_S_ = 2000 cm^−1^) to minimize the non-resonant signal contribution from structures other than pheomelanin. Image stacks are 27 μm thick, with a step size of 1 μm. An exemplary video scrolling through each individual image of the CARS depth stack corresponding to (**b**) can be found in the supplementary materials (Supplementary Video S1). Note that an out-of-focus hair (blue arrow) caused a shadow in the trans-illumination image (**a**), whereas CARS is highly depth-resolved; therefore, the hair was not prominently seen in (**b**).

With the evidence that pheomelanin may be selectively visualized in isolated genetically defined cells, we explored the detection and localization of pheomelanin within intact mouse skin. *Ex vivo* ear skin from C57BL/6 (*Mc1r*^*e/e*^, *Tyr*^+/+^) red-haired mice proved to be an advantageous sample, as brightfield images acquired alongside CARS could be used to verify the location of melanin within the tissue. Under the eyepiece, pheomelanotic cells in the thin ear skin appeared brown-red, with visible cell bodies and dendrites (Fig. 2). Individual pheomelanin-containing organelles were distinctly visualized via CARS imaging and co-localized with the pigmented areas observable by brightfield microscopy.

To translate these findings, CARS imaging was carried *in vivo* on red-haired mouse (*Mc1r*^*e/e*^, *Tyr*^+/+^) ears. Identical patterns of pheomelanin-rich cells were found (Supplementary Fig. S3). Importantly, these *ex vivo* and *in vivo* experiments were repeated for both control albino-red (*Mc1r*^*e/e*^, *Tyr*^*c/c*^) and albino (*Mc1r*^+/+^, *Tyr*^*c/c*^) mice: no visible pheomelanin signals could be observed in the skin of these animals.

**Figure 3:**
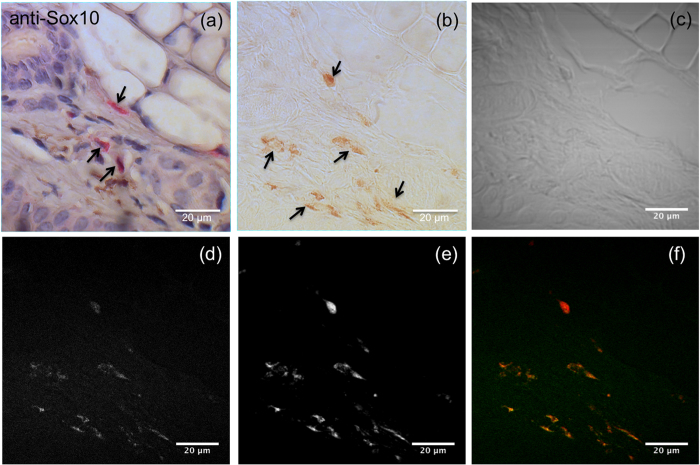
Imaging of pheomelanin stores in a red-haired (C57BL/6 (*Mc1r*^*e*/*e*^, *Tyr*^+/+^) mouse ear section (5 μm thickness). (**a**) Immunohistochemical stain of the mouse ear slide using anti-Sox-10 antibody (counterstain: haematoxylin), revealing melanocytes in red, some of which are indicated by black arrows for added clarity. (**b**) Image of an adjacent unstained 5 μm thick slide under brightfield illumination, revealing pheomelanin-rich deposits (shown by black arrows) consistent with the localization of the melanocytes in the adjacent slide shown in (**a**). (**c**) Trans-illumination image of the unstained slide shown in (**b**) acquired with the pump beam set to 861 nm. (**d**) CARS and (**e**) SFA images of the unstained slide shown in (**b**), revealing bright granular signals from pheomelanotic stores consistent with positive staining in (**a**) and pigmented areas in (**b**). (**f**) False-colour overlaid image of (**d**) in green and (e) in red. A CARS image acquired with the pump beam set to 871 nm (ω_P_ − ω_S_ = 1866 cm^−1^) was subtracted from the image acquired with the pump beam set to 861 nm (ω_P_ − ω_S_ = 2000 cm^−1^) to minimize the non-resonant signal contribution from structures other than pheomelanin.

To confirm these measurements and positively identify the observed pheomelanin-rich cells as melanocytes, thin sections (5 μm in thickness) were prepared from *Mc1r*^*e/e*^, *Tyr*^+/+^ red-haired mouse ears for histology and immunohistochemistry (IHC). As the red fluorescence from eosin was found to interfere with the CARS signal, immunostained slides where accompanied by an adjacent unstained slide for coherent Raman imaging. As this alternative slicing method was used, image features will not align precisely, but they are nevertheless relatively close to one another with a high degree of spatial consistency. Sry-related HMG-BOX gene 10 (Sox10) is a nuclear transcription factor that participates in neural crest development and in the specification and differentiation of cells of the melanocytic lineage. Sox10 is expressed in Schwann cells and melanocytes/melanomas^10^, making it a specific marker for dermal melanocytes. Figure 3(a) shows IHC staining for Sox10 (EP268, Cell Marque), where pheomelanin-containing melanocytes were found to be Sox10 positive. Pheomelanin stores inside the red-haired mouse skin can also be seen under brightfield illumination in the adjacent unstained slide, as shown in Fig. 3(b). When imaging the same unstained slide with CARS targeting the pheomelanin vibrational band, bright pheomelanin signals were found corresponding to the pigmented areas seen under brightfield and in the Sox10 positive areas via IHC. A separate experiment was also conducted to image a different mouse ear sample without IHC staining, such that CARS imaging can be performed directly on the haematoxylin-stained slice. As shown in Supplementary Fig. S4, pheomelanotic stores are observed as bright granular structures, consistent with imaging data shown in Fig. 3.

In order to further cross-validate the pheomelanin signals from the CARS images, we also simultaneously performed sum frequency absorption (SFA) imaging in a multimodal configuration, where SFA microscopy is an imaging toolkit that enables the visualization of light absorbing molecules like pheomelanin^11^,^12^. This optical process is characterized by a molecule’s ability to simultaneously absorb two photons without the necessary involvement of an intermediate real energy level between the ground state and the final excited state. The signal was confirmed to arise from sum frequency absorption and not stimulated Raman scattering (SRS) by tuning the pump wavelength across the expected pheomelanin Raman band. As no variation in signal intensity was observed, the signal was determined to arise solely from absorption contrast. As shown in Fig. 3(e) and (f), a one-to-one correlation can be found between CARS and SFA images, indicating that pheomelanin within melanocytes can indeed be well visualized using CARS microscopy. It should be noted that the overlap of the CARS and SFA images with Fig. 3(a) is not perfect, as each tissue slice is 5 μm thick. Moreover, the signal-to-noise ratio in the image shown in Fig. 3(d) is reduced compared to that obtained in tissue due to the configuration of the imaging system: as the position of the CARS detector is in the epi-direction, the collected signal depends in part on back-scattering in order to redirect the forward-generated anti-Stokes light back into the microscope objective. Considering the 5 μm thickness of the tissue slice, the CARS image quality in this scenario does not benefit from the scattering properties of thick biological samples that would otherwise generate a much stronger signal^13^. It is also possible that the actual amount of pheomelanin in thin tissue slices is slightly diminished relative to intact tissue as a result of the frozen section preparation itself. Nevertheless, the consistency in spatial localization between the signals observed in Fig. 3(d) and (e) indeed supports the ability of CARS microscopy to specifically visualize pheomelanin in a label-free manner.

### Pheomelanin detection in human amelanotic melanoma

**Figure 4:**
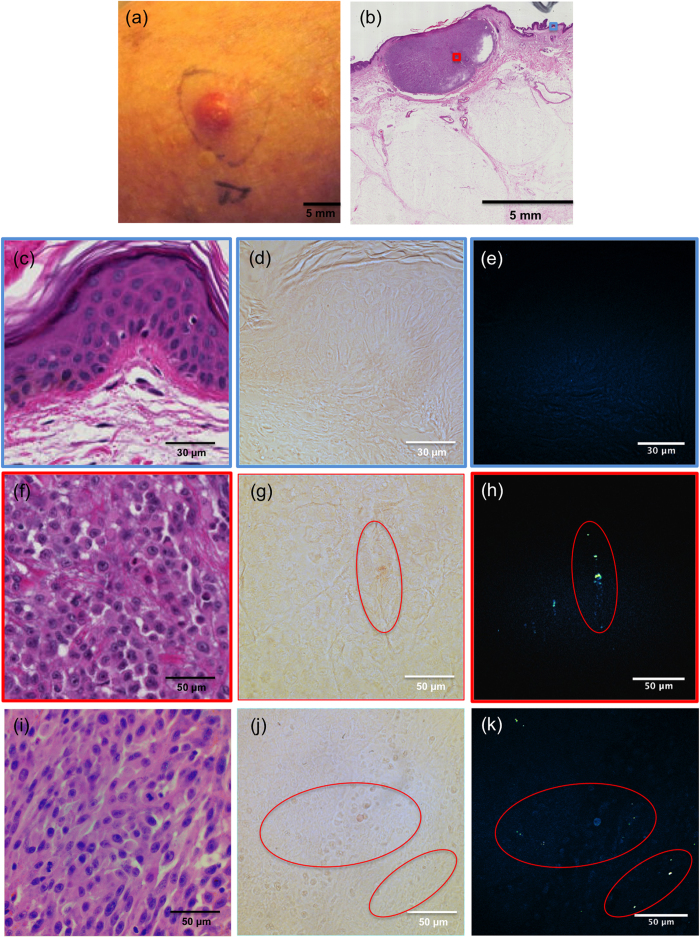
Imaging of human amelanotic melanoma. (**a**) Clinical photograph of one amelanotic melanoma lesion. (**b**) H&E stain of the patient slide (10X magnification). (**c**) Perilesional skin showing normal architecture of both epidermis and dermis. (**d**) Brightfield trans-illumination image acquired from the microscope eyepiece from the perilesional area. (**e**) CARS image of the same perilesional area compared to (**d**) (image acquired with pump beam wavelength at 841 nm (ω_P_ − ω_S_ = 2275 cm^−1^) was subtracted from the image acquired with pump beam wavelength at 855 nm (ω_P_ − ω_S_ = 2081 cm^−1^) to minimize the non-resonant background from structures other than pheomelanin). (**f**) View of the amelanotic melanoma area showing high density of cells with no obvious sign of melanin. (**g**) Brightfield trans-illumination image acquired from the microscope eyepiece from an unstained slide of the melanoma area, showing slightly pigmented granular structures (red circle). (**h**) CARS image of the same tumour area compared to (**g**), with the same settings as for (**e**). Saturated bright pheomelanin signals were found (red circle) corresponding to the minimally pigmented region shown in (**g**). (**i,j,k**) Respectively H&E, trans-illumination, and CARS images of the tumour area of slides from a second amelanotic melanoma patient. Strong pheomelanin signals were again observed (red circles).

With evidence showing that pheomelanin can be noninvasively visualized in synthetic, *in vitro*, *ex vivo*, and *in vivo* settings, we asked whether pheomelanin could be visualized in human specimens, specifically in the context of amelanotic melanoma. This melanoma subtype is characterized by its lack of traditional brown-black pigmentation upon visual inspection, typically presents as a raised lesion on the skin that is reddish in colour, and can easily be misdiagnosed due to its lack of dark pigmentation^14^. While the terminology seemingly implies the lack of any pigmentation, it is unclear whether some amelanotic melanoma lesions actually do harbour stores of pheomelanin that are simply indistinguishable from surrounding healthy skin. Moreover, recent studies^4^ have indicated the oncogenic potential of pheomelanin in red-haired and fair-skinned backgrounds, raising the possibility that at least some seemingly unpigmented melanoma lesions may contain pheomelanin, but are undetectable to the naked eye. CARS imaging and spectroscopic measurements were performed on fixed and unstained sections (10 μm in thickness) from three cutaneous amelanotic melanoma specimens. One such lesion can be seen in Fig. 4(a) along with its corresponding H&E cross-section. Inspection of normal, healthy perilesional skin with CARS (Fig. 4(c–e)) did not reveal detectable pheomelanin. Within the amelanotic melanoma lesion, however, strong pheomelanin CARS signals were observed indicating a high density of pheomelanin (representative images shown in Fig. 4(f–h)). Pheomelanin was ultimately detected in all three patient samples imaged, with representative images from a second patient shown in Fig. 4(i–k). This suggests that amelanotic melanoma lesions can indeed contain dense stores of pheomelanin, which may be detected noninvasively using CARS imaging.

It should be emphasized that the number of human samples surveyed in this work was limited to three, corresponding to the total number of amelanotic melanoma tissues available for study. In order to truly determine the incidence of pheomelanotic melanoma lesions among those diagnosed as “amelanotic”, this proof-of-concept work will have to be expanded into a broader study: one where multiple CARS image stacks taken from a large patient cohort will be processed and statistically analysed to gain insight that can impact clinical practice.

Indeed, it has been proposed that an intrinsic pheomelanin pathway may represent a biologically significant contributor to melanomagenesis^4^. Building a complete understanding of this contribution, however, has been hampered by an inability to spatially map and quantify pheomelanin within skin. While the detection and differentiation of melanins has been accomplished using pump-probe microscopy^12^,^15^,^16^,^17^, measurements of pigments’ excited state can be complicated by compounds in the local environment (e.g. metals such as iron). Moreover, distinguishing melanin subtypes using pump-probe microscopy requires the acquisition of so-called “delay stacks”, where images are sequentially acquired with varying time delay between the pump and probe beams in order to obtain transient absorption traces on a pixel-by-pixel basis. From there, melanin subtypes can be differentiated using post-processing techniques such as principal component^12^ or phasor analyses^17^. However, each frame typically requires an acquisition time of several tens of seconds, leading to a total acquisition period ranging from 5 to 15 minutes for a single delay stack^17^. On the other hand, CARS imaging offers a specific, direct, and facile route for the detection of pheomelanin *in vivo* in real-time, since the detected signal arises from the generation of a new colour of light mediated by the pheomelanin pigment itself. In this regard, CARS microscopy offers many of the same benefits as multiphoton microscopy: the signal can be readily detected using an appropriate set of laser sources and optical filters, and has additionally been shown to operate up to video-rate imaging speeds^18^.

Prior investigation of melanin pigments using spontaneous Raman spectroscopy found pheomelanin to harbour three broad vibrational peaks at 500, 1490, and 2000 cm^−1^, while eumelanin was shown to have peaks at 500, 1380, and 1580 cm^−1 ^5,6. The first two peaks of pheomelanin as well as all those of eumelanin are located within the fingerprint region of the Raman spectrum; they are therefore unlikely to offer a straightforward route to highly specific detection of melanin pigment subtypes in the native context of human skin, as vibrational peaks from other chemical species would result in spectral interference. However, in the particular case of pheomelanin, the broad vibrational peak of interest in this study is located in the 2000–2100 cm^−1^ range^5^,^6^, well within the so-called “silent region” of the Raman spectrum of biological samples^13^. This spectral region, which ranges approximately from 1800 to 2700 cm^−1^, is devoid of vibrational peaks that may arise from other endogenous biochemical moieties, making this particular band of pheomelanin an ideal target for future clinical CARS imaging with minimal interference from other compounds. It is worth nothing that the use of deuterated chemical groups as Raman reporters may interfere with pheomelanin imaging, as the carbon-deuterium vibrational band is located around 2200 cm^−1^. In this highly special case, the use of hyperspectral coherent Raman imaging may be used to distinguish the peaks in the event of signal overlap between pheomelanin and deuterated probes. It should also be noted that the unlikely possibility of interference from eumelanin at the 2000–2100 cm^−1^ band was also investigated in the context of this study. Importantly, no coherent Raman signals could be detected from eumelanin within the pheomelanin Raman band range (data not shown), further strengthening the specificity of CARS for the direct visualization of pheomelanin without interference from its eumelanin counterpart.

In this work, the use of multiple genetically controlled systems, including the *Mc1r* mutant mice and their corresponding albino/red-haired controls (*Mc1r*^*e/e*^, *Tyr*^*c/c*^), enabled confirmation of the CARS signal as specifically arising from pheomelanin since these animal models are incapable of producing eumelanin^4^. Interestingly, the strength of the observed anti-Stokes signal was far greater than anticipated based on the previously published spontaneous Raman data^5^,^6^, indicating the possibility that the observed signal is resonantly enhanced^19^.

Furthermore, for future work in translating this imaging modality for clinical studies, CARS microscopy has even been performed using a single laser source, where one portion of the laser light was used as the pump beam, while the remainder was routed through either a tapered fibre^20^ or a photonic crystal fibre^21^ in order to generate the Stokes beam. This flexibility in terms of optical design in conjunction with the chemical specificity offered by CARS imaging makes it a prime candidate as an imaging tool for future clinical investigations in human patients. In fact, as portable CARS systems are already commercially available^22^, the translation of the presented technology for further investigation in research or clinical settings can be immediate and rapid. We believe this imaging approach will aid in improving our biological understanding of skin changes in the context of pheomelanin, including both UV-induced and UV-independent effects. This imaging approach may also help to refine our understanding of which cutaneous lesions are truly “amelanotic” as opposed to those that may be “pheomelanotic” and currently invisible to the naked eye. Additionally, the method may help to define inhomogeneously coloured melanocytic or non-melanocytic lesions, whose “unpigmented” regions might contain detectable pheomelanin, and represent challenges in defining surgical margins. The ability to identify such lesions non-invasively and non-destructively will enable future studies investigating the impact of pheomelanin on melanoma formation, pathogenesis and metastasis in different human skin types. The prospect of utilizing this method to detect suspicious, pre-malignant lesions in high-risk (red-haired, fair-skinned) individuals, may offer new diagnostic tools able to translate knowledge from basic science to clinical application.

## Materials and Methods

### Mice

Mice with the *Mc1r* frameshift mutant allele (*Mc1r*^*e/e*^) have a phenotype analogous to red-hair/fair-skin in humans (also caused by non-functional alleles of MC1R)^4^. As control, we crossed these mice to the albino allele (*Mc1r*^*e/e*^, *Tyr*^*c/c*^.), which ablates all melanin pigment production. All mouse strains were maintained on the C57BL/6 background.

### CARS microscopy

The CARS microscope was built over a customized confocal microscope (Olympus FV1000, Center Valley, PA, USA), which has an additional laser entry port to accept external light sources. CARS microscopy was performed using a dual output femtosecond pulsed laser system (Spectra-Physics Insight DeepSee, Santa Clara, CA, USA), where the first output is tuneable from 680 to 1300 nm, while the second is fixed at 1040 nm. To achieve CARS imaging at the reported 2000 cm^−1^ band of pheomelanin, the 1040 nm output was chosen as the Stokes beam (ω_S_), while the pump beam (ω_P_) was set to 861 nm and 855 nm (ω_P_ − ω_S_ = 2000 cm^−1^ and ω_P_ − ω_S_ = 2081 cm^−1^, respectively), in order to generate anti-Stokes signals at 735 nm and 726 nm, respectively. A half-wave plate and a polarizer were placed at each of the two laser output ports of the DeepSee system to adjust beam power. To focus the beams onto the sample, a 1.20 NA 60X water immersion microscope objective (Olympus UPLSAPO 60XW, Center Valley, PA, USA) was used. CARS signal was detected in the epi-direction using both a shortpass and a bandpass filter (Chroma ET750sp-2p8 and ET730/40 m, Bellows Falls, VT, USA) placed in front of a thermoelectrically cooled photomultiplier tube (Hamamatsu H7422PA-50, Hamamatsu City, Japan). The sum power of the two beams at the objective was less than 10 mW for all experiments performed in this study. In order to correct for the non-resonant background signal generated in tissues, both resonant and non-resonant CARS images were obtained in order to subtract the non-resonant contribution from the resonant CARS images. To this aim, the pump beam was tuned to either 841 nm or 871 nm (resulting in a Raman shift of 2275 cm^−1^ or 1866 cm^−1^, respectively). In both of these non-resonant imaging settings, the background levels were found to be identical; the choice of tuning the pump beam to 841 nm or 871 nm was therefore simply based on the convenience of the experiment at hand. This correction was not necessary for cellular samples, as the signal detected in the epi-direction contains minimal contribution from back-scattered light and has inherently low non-resonant background^13^.

### SFA microscopy

All SFA imaging experiments were performed simultaneously with CARS imaging on the same system as described above, using the same wavelengths and power levels. However, instead of detecting signal in the epi-direction, the SFA detector was placed downstream of the sample in the trans-direction. The 1040 nm beam was modulated at 20 MHz using an electro-optic modulator (ThorLabs EO-AM-R-20-C2, Newton, NJ, USA), and the modulation transfer from the 1040 nm beam to the pump beam was detected using a photodiode coupled to a lock-in amplifier (APE Lock-In Amplifier, Berlin, Germany). In order to restrict the light incident on the SFA detector to only that of the pump beam, a shortpass filter (ThorLabs FES0950, Newton, NJ, USA) was used to eliminate the 1040 nm light. Ordinarily, this detection setup would be sensitive to stimulated Raman scattering (SRS) signals as well. However, it was found that when imaging pheomelanin, the detected signal remained constant across a range spanning 1866 to 2275 cm^−1^. This observation was additionally confirmed in synthetic pheomelanin samples, which showed the same temporal and wavelength tuning properties as the pheomelanin detected in tissue samples. This suggests that the signals obtained from pheomelanin are primarily a result of its absorptive properties, rather than its intrinsic vibrational modes.

### Preparation of synthetic pheomelanin

Pheomelanin was synthesized following the protocol published by d’Ischia *et al*.^7^. The synthesized pheomelanin was then lyophilized to yield a dense, reddish-brown powder. To emulsify the pheomelanin, a small quantity (~0.1 mg) was placed in a 2 mL microcentrifuge tube, to which 1 mL H_2_O and 0.25 mL hexane were added. The resulting mixture was sonicated for two cycles of three minutes each to generate an emulsion of pheomelanin microparticles. The sample was then sandwiched between a glass slide and a coverslip with an imaging spacer (Grace Bio-Labs Secure-Seal, Bend, OR). To prevent sample evaporation, the edges of the coverslip were sealed to the glass slide with nail polish.

### CARS spectral data acquisition and processing

In order to generate CARS spectra of pheomelanin samples, the pump wavelength was tuned from 841 nm to 871 nm in single nanometre increments. For each wavelength value, 3 images were acquired: the first with only the pump beam, the second with only the Stokes beam, and the third with both. While the first two image sets showed only minimal intensity, they nevertheless corresponded to the weak multiphoton fluorescence of pheomelanin and were thus subtracted from the CARS images to isolate the coherent Raman signal. In order to normalize the CARS signal against a neutral reference, the sample’s glass coverslip was also imaged under the same conditions. Glass produces a non-resonant background signal that is invariant across the spectral range of interest, justifying its use as a reference to compensate for wavelength-dependent intensity variations that may arise from the optical components throughout the imaging system.

In order to generate the spectra, the 31 corrected CARS images were first summed together, and the resulting image was binarized to generate a mask. The mask was then applied to all CARS and glass images in order to isolate the regions corresponding to pheomelanin. The masked CARS images were then divided by the masked glass images on a pixel-by-pixel basis, and the resulting ratiometric values were averaged to obtain a single data point for a given wavelength. This process was iterated across all 31 sampled wavelengths, and the resulting spectra were normalized by the area under the curve and multiplied by a factor of 30 (corresponding to n-1 data points) such that a flat spectral response was centred at a normalized value of 1 across the entire spectral range. The experiment was performed in triplicate, where each individual spectrum is plotted in grey and the mean of the three measurements is plotted in red. As an added control, the analysis was repeated for the image background by inverting the binary mask generated earlier. Given that the image background signal arises from the non-resonant background generated by the water and hexane emulsion, the analysis performed on the background revealed a flat spectrum centred at 1, as expected.

### Melanocyte extraction

Two mouse strains were utilized to isolate neonatal cutaneous melanocytes: 1) *Mc1r*^*e/e*^: K14-SCFTg+^4^; Tyr-CreTg+^8^: B6.Cg-*Gt(ROSA)26Sor*^*tm9(CAG-tdTomato)Hze*^/J (Jackson Laboratory catalogue #007909) and 2) *Mc1r*^*e/e*^: K14-SCFTg+; Tyr-Cre: B6.Cg-*Gt(ROSA)26Sor*^*tm6(CAG-ZsGreen1)Hze*^/J (Jackson Laboratory catalogue #007906) were used as sources of neonatal pheomelanotic cutaneous melanocytes. The K14-SCF transgene mimics human epidermal expression of SCF and enhances the yield of cutaneous melanocytes^4^. Melanocyte targeted fluorescent tags were generated using the constitutive Tyr:Cre transgene^8^. Neonates (day 2) were euthanized. The truncal skin was peeled away and washed twice with PBS containing Pen:Strep and Fungizone. The skin was then placed in a 60 mm tissue culture dish with 3 mL of dispase (25 mg/mL) and placed in a 37 ^o^C, 5% CO_2_ incubator for 2 hours. The epidermal layer was peeled away and incubated in another 60 mm tissue culture dish containing 3 mL of 0.25% trypsin for 15 minutes at 37 ^o^C. The skin tissue was washed in 10 mL of Hams F10 medium containing 10% FBS, ten times. The cells removed using this procedure were then washed and suspended in PBS that contained DNAse I (50 μg/mL) and 5 mM EDTA. The cells were sorted for the highest expression of either tdTomato or ZsGreen in a BD FACSAria II SORP cell sorter. ZsGreen cells were excited with a 488 nm laser and detected with a 525/50 nm bandpass filter; tdTomato cells were excited with a 561 nm laser and detected with a 582/15 nm bandpass filter. To confirm the identity of the melanocyte population, the tdTomato cells in another experiment were counterstained with FITC c-Kit, a surface marker for both melanocytes and mast cells. When imaging FACS-isolated cells using CARS microscopy, the presence of fluorescent proteins in the sample (i.e. tdTomato and ZsGreen) sometimes resulted in a background signal generated via two-photon excitation fluorescence from either or both the pump and Stokes lasers. To correct for this, a raw CARS image was first acquired using both lasers; then, two-photon fluorescence images were acquired by using each laser beam individually. The resulting fluorescence images were then subtracted from the raw CARS image in order to isolate the true CARS signal from the sample.

### Mouse ear imaging

For *ex vivo* mouse ear imaging, the ear tissue was acquired via ear punch (Φ = 3 mm). A commercial hair removal lotion (Nair, Church & Dwight Co., Inc., Princeton, NJ) was used to remove fine hairs from the ear tissue. The sample was then sandwiched between a coverslip and a glass slide, and imaged as described above. For *in vivo* mouse ear imaging, the mice were anesthetized with isoflurane mixed with 0.2 L/min oxygen and 0.8 L/min air via face mask. Fine hairs were removed using the same hair removal lotion as in the *ex vivo* sample. The ear was then fixed onto a coverslip using double-sided tape, and imaged as described above. All studies and procedures involving animal subjects were approved by the Institutional Animal Care and Use Committee of Massachusetts General Hospital, and were conducted strictly in accordance with the approved animal handling protocols.

## Additional Information

**How to cite this article**: Wang, H. *et al*. *In vivo* coherent Raman imaging of the melanomagenesis-associated pigment pheomelanin. *Sci. Rep.* **6**, 37986; doi: 10.1038/srep37986 (2016).

**Publisher's note:** Springer Nature remains neutral with regard to jurisdictional claims in published maps and institutional affiliations.

## Supplementary Material

Supplementary Information

Supplementary Video S1
