# Supplementary material for: In vivo coherent Raman imaging of the melanomagenesis-associated pigment pheomelanin
Source: Sci Rep. 2016 Nov 28;6:37986. doi: 10.1038/srep37986 (PMC5125099; doi:10.1038/srep37986)
Supplement: Supplementary Information [file srep37986-s1.pdf]

Supplementary information

In vivo coherent Raman imaging of the melanomagenesis-associated pigment pheomelanin

Hequn Wang[1], Sam Osseiran[1,2], Vivien Igras[3], Alexander J. Nichols[1,2,4], Elisabeth M. Roider[3], Joachim Pruessner[1], Hensin Tsao[1], David E. Fisher[3*], Conor L. Evans[1,4,5*]

[1]Wellman Center for Photomedicine, Harvard Medical School, Massachusetts General Hospital, 149 13th Street, Charlestown, Massachusetts 02129, USA

[2]Harvard-MIT Division of Health Sciences and Technology, 77 Massachusetts Avenue E25-519, Cambridge, Massachusetts 02139, USA

[3]Cutaneous Biology Research Center, Harvard Medical School, Massachusetts General Hospital, 149 13th Street, Charlestown, Massachusetts 02129, USA

[4]Harvard University Program in Biophysics, Building C2 Room 112, 240 Longwood Avenue, Boston, Massachusetts 02115, USA

[5]Ludwig Center at Harvard, Harvard Medical School, 200 Longwood Avenue, Boston, Massachusetts 02115, USA

* Corresponding authors:

(1) Conor L. Evans, Wellman Center for Photomedicine, Harvard Medical School, Massachusetts General Hospital, 149 13th Street, Charlestown, Massachusetts 02129, USA. E-mail: evans.conor@mgh.harvard.edu

(2) David E. Fisher, Cutaneous Biology Research Center, Harvard Medical School, Massachusetts General Hospital, 149 13th Street, Charlestown, Massachusetts 02129, USA. E-mail: dfisher3@mgh.harvard.edu

To first verify that the developed CARS imaging system can visualize pure pheomelanin molecules, we followed a well-established protocol and synthesized pheomelanin from L-Dopa and tyrosine in the presence of cysteine.[1] To favour the formation of microparticles, the synthetic pheomelanin was mixed with a 4:1 ratio of water to hexanes and sonicated for 3 minutes. CARS microscopy was then performed using a dual output femtosecond pulsed laser system (Insight DeepSee, Spectra-Physics, Santa Clara, CA), where the first output is tuneable from 680 to 1300 nm while the second is fixed at 1040 nm. To achieve CARS imaging at the reported 2000 cm^{-1} band of pheomelanin, the 1040 nm output was chosen as the Stokes beam, while the tuneable output was set to 861 nm as the pump beam. Not only was a strong CARS signal observed from the pheomelanin aggregates at the 2000 cm^{-1} band, but the signal had a strong spectral dependence, as expected from a vibrational resonance. To quantify this dependence, a CARS spectrum was collected showing a broad peak centred around 2081 cm^{-1}, which is consistent with published results using Raman spectroscopy.[2,3] Figure S1(a) and (b) show CARS and trans-illumination images of synthetic pheomelanin aggregates, respectively. Circular features are hexane bubbles, whereas the bright irregular structures correspond to pheomelanin. To account for the spectral dependence of optical aberrations, power levels, and detector sensitivities, the non-resonant signal generated by a glass coverslip was measured and served as a frequency-independent reference for synthetic pheomelanin spectral data processing. Figure S1(c) shows the CARS spectrum of the synthetic pheomelanin, referenced to glass and normalized by the area under the curve. The measured CARS spectrum of pheomelanin is centred around 2081 cm^{-1}, which is slightly shifted from the 2000 cm^{-1} peak observed in published Raman spectra of pheomelanin.[2,3] This shift can be attributed to the sensitivity of the spectral peak position to the pheomelanin species considered. Indeed, this resonant peak was shown to range from 1900 to 2300 cm^{-1}, depending on the animal species from which the pheomelanin-containing samples were obtained.[3]

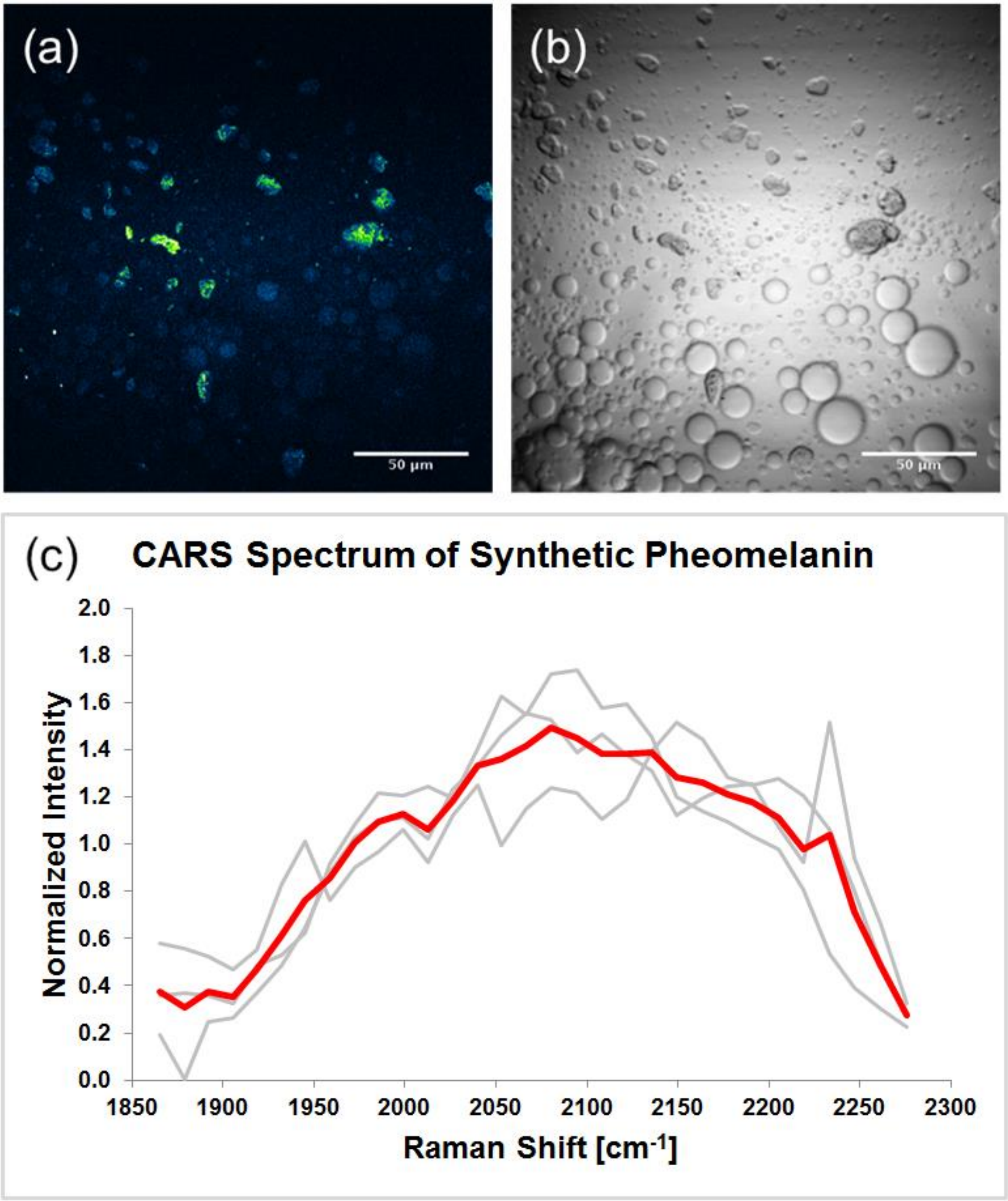

Figure S1. Synthetic pheomelanin in a 4:1 water:hexane emulsion. (a) CARS image acquired at $\omega_P - \omega_S$ = 2000 cm^{-1}. Pheomelanin is observed as small, insoluble aggregates with bright CARS intensity. The surrounding water/hexane bubbles show low-level non-resonant CARS background. (b) Trans-illumination image acquired with the 861 nm pump beam. (c) CARS spectrum of synthetic pheomelanin referenced to the wavelength-independent non-resonant signal from a glass coverslip, normalized by area under the curve. Three synthetic pheomelanin samples were measured, with each spectrum shown in grey; the curve in red corresponds to the mean of the three measurements.

Next, as shown in Fig. S2, melanocytes isolated from albino-red mice did not show the punctate/vesicular features that were observed in the melanocytes extracted from the red-haired mice. The signals detected from the CARS channel contains mainly the fluorescence generated from the pump beam (λ = 861 nm), such that the true CARS signals can be extracted by subtracting out the fluorescence contributions from the pump and Stokes beams as shown in Fig. S2(f), which indicates absence of any vibrational signatures of pheomelanin due to the lack of pigment production in the albino-red mice.

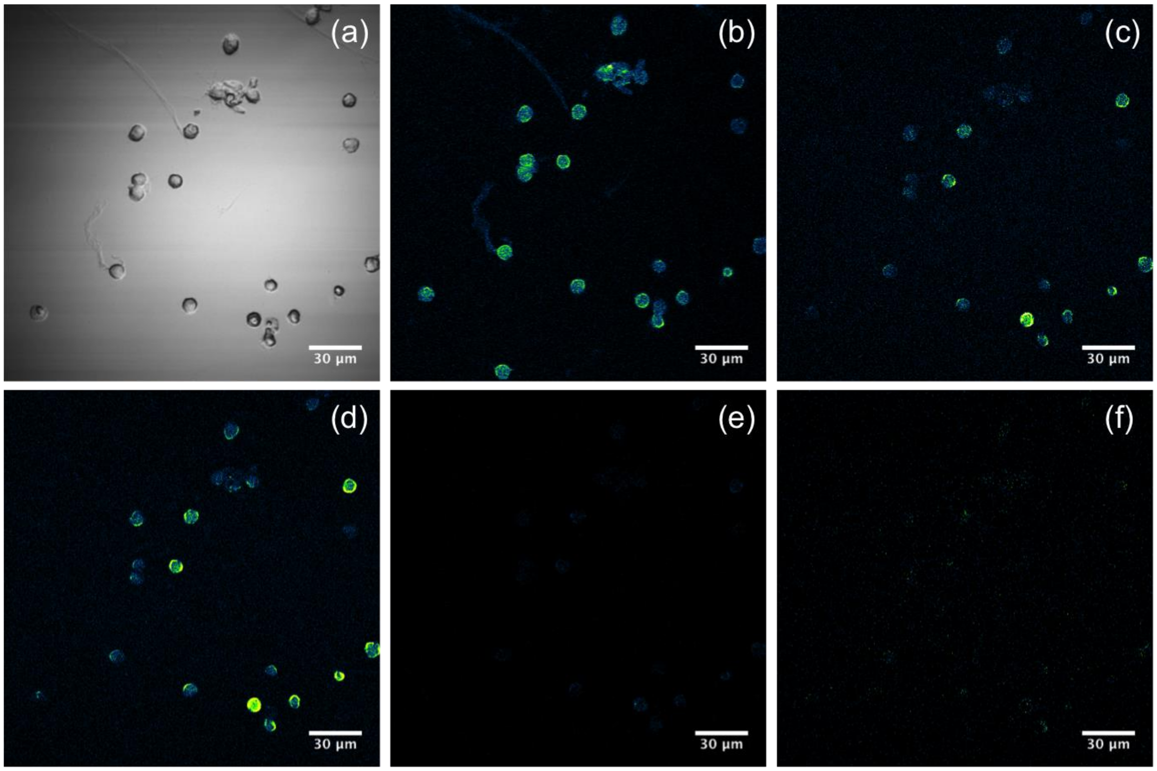

Figure S2. Melanocytes isolated from albino-red C57BL/6 (*Mc1r$^{e/e}$ Tyr$^{c/c}$*) mice exhibit negligible CARS signal at $\omega_P - \omega_S = 2000$ cm^{-1}. (a) Trans-illumination image acquired with the 861 nm pump beam. (b) Confocal fluorescence image of tdTomato. (c) False colour CARS image acquired with pump (λ_P = 861 nm) and Stokes beams (λ_S = 1040 nm). (d) False colour image acquired only using the pump beam, showing prominent two-photon fluorescence signals from tdTomato. (e) False colour image acquired only using the Stokes beam, showing weak two-photon fluorescence signals from tdTomato. (f) False colour image illustrating the true CARS signal obtained by subtracting tdTomato fluorescence in (d) and (e) from the raw CARS image in (c).

Next, pheomelanin stores within the red-haired mouse ear *in vivo* show distinctively bright CARS signals with high contrast against the non-resonant background (Fig. S3), in strong agreement with the *ex vivo* mouse ear imaging results (Fig. 2).

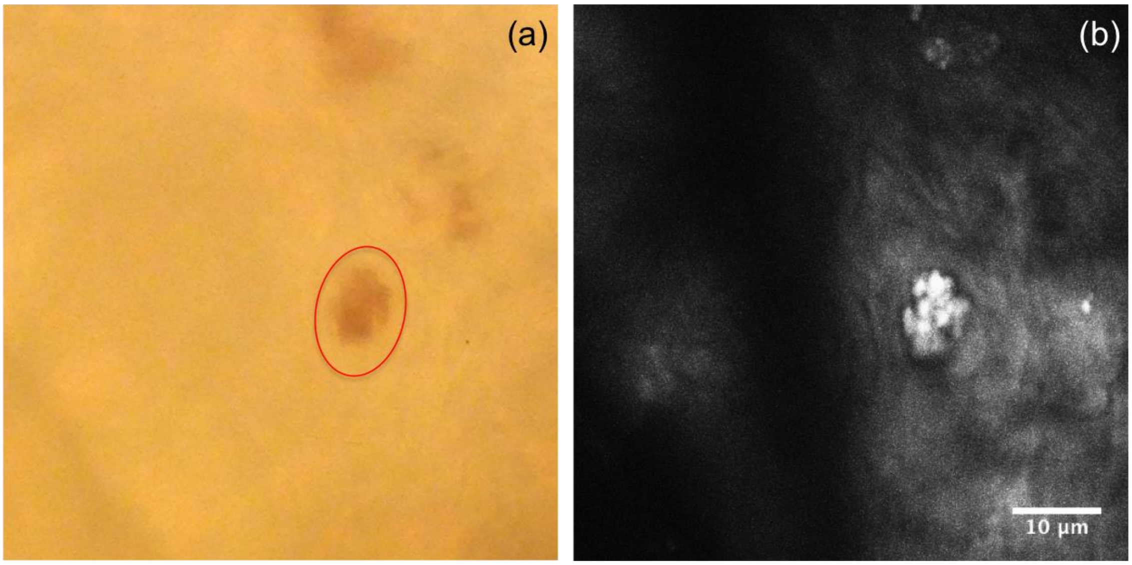

Figure S3. Imaging of pheomelanin stores in a red-haired mouse ear *in vivo*. (a) Brightfield trans-illumination image acquired from the microscope eyepiece. (b) Maximal projection view of CARS image stack of the mouse ear, showing bright signals from pheomelanin (red circle). A CARS image stack acquired with the pump beam set to 871 nm ($\omega_P - \omega_S$ = 1866 cm^{-1}) was subtracted from the image stack acquired with pump beam set to 861 nm ($\omega_P - \omega_S$ = 2000 cm^{-1}) to minimize the non-resonant signal contribution from structures other than pheomelanin. The image stack in (b) is 15 μm thick, with a step size of 1 μm.

Finally, as red fluorescence from eosin was found to interfere with the CARS signal, the red-haired mouse ear was stained with haematoxylin only. The structure of the mouse ear can be well visualized, with pheomelanin stores and associated melanocytes appearing as light brown pigmented clusters in brightfield images (Fig. S4(a) and (b)). The melanocytes in the mouse ear are scattered below the dermal-epidermal junction (DEJ) and within the dermis, clustered around sebaceous glands at the base of hair follicles throughout the ear tissue. Bright punctate signals from one such melanocyte and its pheomelanin stores were observed in CARS microscopy (Fig. S4(c)).

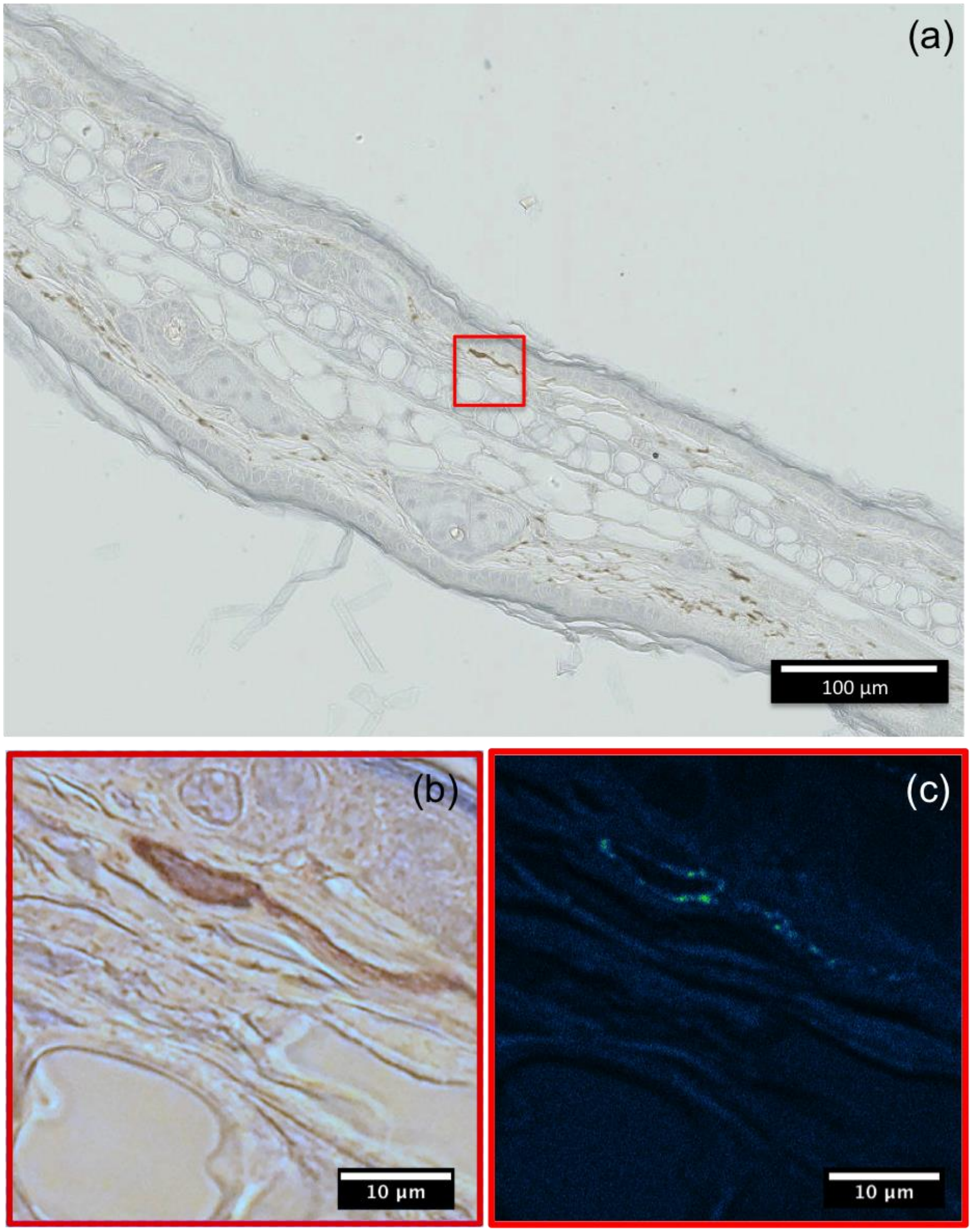

Figure S4. Imaging of pheomelanin stores in a red-haired mouse ear section (5 µm thickness). (a) Brightfield trans-illumination image of the haematoxylin-stained mouse ear slide. (b) Magnified view of the area marked by a red square in (a). Pheomelanotic stores are seen as slightly pigmented granules. (c) CARS image of the same field of view as (b), showing bright granular signals from pheomelanotic stores corresponding to the pigmented areas in (b). A CARS image acquired with the pump beam set to 871nm ($\omega_P - \omega_S$ = 1866 cm^{-1}) was subtracted from the image acquired with the pump beam set to 861 nm ($\omega_P - \omega_S$ = 2000 cm^{-1}) to minimize the non-resonant signal contribution from structures other than pheomelanin.

Supplementary Video S1. Depth stack of CARS images from mouse ear, showing bright granules from the pheomelanin stores within melanocytes and at the base of the hair follicle. A CARS image stack acquired with the pump beam set to 871nm ($\omega_P - \omega_S = 1866\ cm^{-1}$) was subtracted from the image stack acquired with the pump beam set to 861 nm ($\omega_P - \omega_S = 2000\ cm^{-1}$) to minimize the non-resonant signal contribution from structures other than pheomelanin. The image stack is 27 μm thick, with a step size of 1 μm.

References

1 d'Ischia, M. *et al.* Melanins and melanogenesis: methods, standards, protocols. *Pigment cell & melanoma research* **26**, 616-633 (2013).

2 Galván, I. *et al.* Raman spectroscopy as a non-invasive technique for the quantification of melanins in feathers and hairs. *Pigment Cell & Melanoma Research* **26**, 917-923 (2013).

3 Galván, I., Jorge, A., Solano, F. & Wakamatsu, K. Vibrational characterization of pheomelanin and trichochrome F by Raman spectroscopy. *Spectrochimica Acta Part A: Molecular and Biomolecular Spectroscopy* **110**, 55-59, doi:10.1016/j.saa.2013.03.027 (2013).
